# Adaptive Feedback Compensation Algorithm for Quantum Random Number Generators

**DOI:** 10.3390/e27080860

**Published:** 2025-08-14

**Authors:** Wei Deng, Kun Chen, Fei Hua, Jing Cheng, Banghong Guo, Huanwen Xie

**Affiliations:** 1 Guangdong Provincial Key Laboratory of Nanophotonic Functional Materials and Devices, School of Optoelectronic Science and Engineering, South China Normal University, Guangzhou 510006, China; 13049661600@163.com (W.D.);; 2 Guangdong Provincial Key Laboratory of Quantum Engineering and Quantum Materials, School of Optoelectronic Science and Engineering, South China Normal University, Guangzhou 510006, China; 3 National Quantum Communication (Guangdong) Co., Ltd., Guangzhou 510700, China

**Keywords:** adaptive feedback compensation, quantum random number generator, post-processing, dynamic parameter adjustment

## Abstract

As a core component in quantum cryptography, Quantum Random Number Generators (QRNGs) face dual critical challenges: insufficient randomness enhancement and limited compatibility with post-processing algorithms. This study proposes an Adaptive Feedback Compensation Algorithm (AFCA) to address these limitations through dynamic parameter feedback and selective encryption strategies. The AFCA dynamically adjusts nonlinear transformation intensity based on real-time statistical deviations, retaining over 50% of original bits while correcting local imbalances. Experimental results demonstrate significant improvements across QRNG types: the Monobit Test *p*-value for continuous QRNGs increased from 0.1376 to 0.9743, and the 0/1 distribution deviation in discrete QRNGs decreased from 7.9% to 0.5%. Compared to traditional methods like von Neumann correction, AFCA reduces data discard rates by over 55% without compromising processing efficiency. These advancements provide a robust solution for high-security quantum communication systems requiring multi-layered encryption architectures.

## 1. Introduction

Quantum Random Number Generator (QRNG) [1,2,3] serves as a pivotal component in quantum information technology, with its inherent unpredictability and information-theoretic security providing physical-layer guarantees for high-security applications such as quantum key distribution and military encryption [4,5]. However, raw QRNG sequences frequently exhibit statistical deviations due to non-ideal characteristics of quantum entropy sources (e.g., thermal drift and detector dead-time effects), leading to frequency balance deviations. Contemporary post-processing algorithms face three critical challenges: (1) existing methods (e.g., hash functions [6], von Neumann correction [7]) struggle to rectify biases while preserving original sequence features; (2) insufficient algorithmic compatibility in multi-stage post-processing architectures restricts encryption flexibility and anti-attack capabilities [8,9,10]; and (3) inherent trade-offs between efficiency and security in conventional approaches, exemplified by von Neumann correction requiring over 70 percent data discard in specific cases, while SM3 Cryptographic Hash Algorithm (SM3) [11] enhances randomness at the expense of completely erasing quantum entropy source characteristics.

Classical post-processing algorithms predominantly rely on static mechanisms: SM3 achieves global confusion through hash transformations, SM4 Block Cipher Algorithm (SM4) enhances nonlinearity via fixed S-box substitutions [12,13,14], and von Neumann correction filters valid bits using deterministic rules. Research indicates that static algorithms tend to induce over-correction or under-correction in specific scenarios, resulting in suboptimal equilibrium between randomness optimization and computational resource allocation. Consequently, the existing research gap lies in the absence of a post-processing solution capable of precise deviation correction through dynamic feedback mechanisms while maintaining QRNG sequence characteristics and multi-stage cryptographic architecture compatibility. Recent advancements in QRNG design have also explored physical-layer optimizations to mitigate inherent biases, such as Photon Variance QRNG (PV-QRNG) schemes leveraging quantum entanglement properties [15,16]. These approaches aim to minimize post-processing requirements by enhancing raw output uniformity at the entropy source level.

This study proposes an Adaptive Feedback Compensation Algorithm (AFCA) to establish a dynamic, lightweight, and multi-stage compatible post-processing framework. The core design objectives are threefold: (i) implementing continuous adjustment from “strong intervention” to “weak intervention” through real-time statistical parameters (*β*_i_/*α*_i_) feedback mechanisms; (ii) employing selective encryption strategies (Strategy A/B/C) that preserve raw data in high-entropy regions while activating S-box substitutions and dual-inversion transformations exclusively in significant deviation areas, achieving efficiency-feature preservation balance; and (iii) designing modular processing units compatible with SM4, constructing multi-level defense architectures resistant to side-channel attacks.

The paper is organized as follows: Section 2 elaborates the AFCA design principles, including dynamic parameter feedback models, hybrid transformation strategies, and operational examples. Section 3 validates algorithm effectiveness through comparative experiments using raw/processed sequences from continuous/discrete QRNGs. Section 4 benchmarks performance against von Neumann correction and SM3 hash-based algorithms. Section 5 gives the conclusion. Appendix B provides operation examples and result analyses of the algorithm, demonstrating the beneficial effects of the combination of AFCA and SM4, thereby proving the compatibility of the algorithm.

## 2. Algorithm Design and Implementation

The Adaptive Feedback Compensation Algorithm, as a category of block cipher, employs a 128-bit block processing mechanism. This cryptographic technique demonstrates superior dynamic equilibrium capabilities, effectively preserving original sequence integrity while optimizing 0/1 balance. The algorithm proves particularly suitable for implementation, testing, and application in random number products, fulfilling the compound requirements of modern cryptographic application scenarios.

The encryption process implements a two-phase dynamic control strategy: During the initialization phase, the system generates dynamic parameters through analysis of plaintext characteristics, which subsequently guide encryption path selection in real-time. In the core processing phase, a substitution layer comprising four S-boxes collaborates with a linear transformation layer based on 8/16-bit inversion operations to achieve data confusion. Through its operational logic and architectural design, AFCA enhances randomness while retaining critical features of the original sequence, thereby ensuring high integrity preservation during cryptographic transformation. This mechanism provides an encryption solution that balances randomness enhancement with data characteristic preservation for random number applications.

### 2.1. Key Parameters and Transformations

This section elaborates on the critical parameters and transformation operations within the Adaptive Feedback Compensation Algorithm, which constitute the core components of the algorithm. Specifically, it details: (1) the sequence representations of input plaintext and output ciphertext; (2) computational methods for adaptive parameters; (3) architecture of the nonlinear transformation τ with its S-box design; and (4) implementation mechanisms of linear transformation L. These parameters and transformations are engineered to dynamically adjust encryption strategies, ensuring effective enhancement of data balance while preserving partial features of raw data. The complete encryption workflow will be systematically explained in Section 2.2, with all relevant nomenclature and definitions provided in Table 1.

1. Sequence Representation:

The input plaintext with a bit-length of 128 is represented as $X\;=\;(X_{0},X_{1},\ldots ,X_{7})\in {\left(Z_{2}^{16}\right)}^{8}$, where each $X_{i}\;=\;\left(x_{i0},x_{i1}\right)\in {\left(Z_{2}^{8}\right)}^{2}\;\mathrm{for}\;i\;=\;0,1,\ldots ,7$. The output ciphertext with a bit-length of 128 is denoted as $Y\;=\;(Y_{0},Y_{1},\ldots ,Y_{7})\in {\left(Z_{2}^{16}\right)}^{8}$, where each $Y_{i\;}\;=\;\left(y_{i0},y_{i1}\right)\in {\left(Z_{2}^{8}\right)}^{2}\;\mathrm{for}\;i\;=\;0,1,\ldots ,7$. Here, $X_{i},Y_{i}(i\;=\;0,1,\ldots ,7)$ are defined as 16-bit words. The adaptive parameters $\alpha \;=\;(\alpha_{0},\alpha_{1},\ldots ,\alpha_{7})\;\mathrm{and}\;\beta \;=\;(\beta_{0},\beta_{1},\ldots ,\beta_{7})$ are utilized for frequency-adaptive compensation.

2. Adaptive Parameters *α* and *β*:

Given an input $\left(X_{0},X_{1},\ldots ,X_{7}\right)\in {\left(Z_{2}^{16}\right)}^{8},\;i\;=\;0,1,\ldots ,7$, the adaptive parameters $\alpha \;=\;(\alpha_{0},\alpha_{1},\ldots ,\alpha_{7})$ and $\beta \;=\;(\beta_{0},\beta_{1},\ldots ,\beta_{7})$ are computed as follows, where *sum*(*A*) denotes the sum of all bit elements in *A*:

(1) $$\beta_{i+1}=8-sum(X_{i+1})+\beta_{i}-\alpha_{i},i=0,1,\ldots ,7$$

(2) $$\beta_{0}=8-sum(X_{0})$$

(3) $$\alpha_{i}=\left\{\begin{array}{ll} 4, & \mathrm{if}\;8\geq \beta_{i}\geq 4\\ \beta_{i}, & \mathrm{if}\;3\geq \beta_{i}\geq -3\\ -4, & \mathrm{if}\;-4\geq \beta_{i}\geq -8 \end{array}\right.\;\;i=0,1,\ldots ,7$$

Example: For input $X_{0}$ = 0010 1110 0000 1101 and $X_{1}$ = DFEF, Equations (1)–(3) yield: $\beta_{0}$ = 1, $\alpha_{0}$ = 1; $\beta_{1}$ = −6, $\alpha_{1}$ = −4.

3. Nonlinear Transformation τ:

The S-box functions as a nonlinear substitution table within cryptographic algorithms, mapping input data to output data to enhance randomness and resistance against attacks. It operates as a fixed 8-bit input to 8-bit output permutation. The nonlinear transformation τ is composed of four S-boxes. For different parameter values ($\alpha_{i}$ = ±1, ±2, ±3, ±4), the S-boxes adopt differentiated configurations to achieve dynamic nonlinear intensity regulation. Specific configuration data for these S-boxes are provided in Appendix A Table A1, Table A2, Table A3 and Table A4. For an input vector $A\;=\;(a_{0},a_{1}{,a}_{2}{,a}_{3})\in {\left(Z_{2}^{8}\right)}^{4}$, the output vector $B\;=\;(b_{0},b_{1}{,b}_{2}{,b}_{3})\in {\left(Z_{2}^{8}\right)}^{4}$ is defined by Equation (4):

(4) $$(b_{0},b_{1},b_{2},b_{3})=\tau (A)=\left(\begin{array}{c} Sbox(a_{0}),Sbox(a_{1}),Sbox(a_{2}),Sbox(a_{3}) \end{array}\right)$$

Example: When $\alpha_{i}$ = ±2, for an input value 07, the S-box output is determined by indexing the 0th row and 7th column in the corresponding table, yielding *Sbox* (07) = F8.

4. Linear Transformation *L.*

The linear transformation *L* represents a 16-bit bitwise negation operation, while *l* denotes an 8-bit bitwise negation operation. For an input $C\;=\;(c_{0},c_{1})\in {{(Z}_{2}^{8})}^{2}$ is defined by Equations (5) and (6):

(5) $$D=(d_{0},d_{1})=L(C)=\left(\begin{array}{c} l(c_{0}),l(c_{1}) \end{array}\right)$$

(6) $$D=C⊕FFFF=((c_{0}⊕FF),(c_{1}⊕FF))$$

Example: For the input 1011 1110 0000 1000, applying Equations (5) and (6) yields the negated output 0100 0001 1111 0111.

### 2.2. Encryption Process

The AFCA comprises up to 16 adaptive parameter operations, 8 decision operations, 8 nonlinear transformations *τ*, and 16 linear transformations *L*. Its encryption workflow achieves a balance between enhanced randomness and data integrity. The core logic can be summarized as a three-phase iterative process: Dynamic parameter computation, Encryption strategy selection, and Linear-nonlinear transformations.

1. Dynamic Parameter Computation:

The algorithm first computes the adaptive parameters *α_i_* and *β_i_* based on the bitwise characteristics of the input plaintext group *X_i_*, using Equations (1)–(3). The recursive calculation mechanism for *β_i_* propagates the statistical characteristics of the preceding group to subsequent groups, establishing a feedback compensation mechanism. The parameter *α_i_* is piecewise constrained according to the value range of *β_i_*, providing a quantitative basis for selecting subsequent encryption strategies.

2. Encryption Strategy Selection:

Let the plaintext input be $X_{i}\;=\;\left(x_{i0},x_{i1}\right)\in {\left(Z_{2}^{8}\right)}^{2}$, and the ciphertext output be $Y_{i}\;=\;\left(y_{i0},y_{i1}\right)\in {\left(Z_{2}^{8}\right)}^{2}$, where *i* = 0, 1, …, 7. Based on the value range of *α*_i_, the algorithm employs three distinct encryption strategies (Equations (7)–(9)):

- Strategy A (*α_i_* = 0):
  (7) $$Y_{i}=(y_{i0},y_{i1})=(x_{i0},x_{i1}),i=0,1,\ldots ,7$$

Directly outputs the original group $Y_{i}$ = $X_{i}$. This strategy is applied when the input sequence already exhibits high randomness, avoiding redundant computations.

- Strategy B ($1\;\leq {\;\alpha }_{i\;}\leq \;4$):
  (8) $$Y_{i}=(y_{i0},y_{i1})=(x_{i0},Sbox(x_{i1})),i=0,1,\ldots ,7$$

Applies S-box substitution to the latter half-word to enhance local randomness via nonlinear transformation, while preserving the features of the former half-word.

- Strategy C ($-4\;\leq {\;\alpha }_{i}\;\leq \;-1$):
  (9) $$Y_{i}=(y_{i0},y_{i1})=L(l(x_{i0}),Sbox(l(x_{i1}))),i=0,1,\ldots ,7$$

First performs a linear negation transformation on the former half-word, applies S-box substitution to the negated latter half-word, and finally executes a global negation. This strategy shares S-box tables with Strategy B through dual negation, optimizing storage requirements and computational efficiency.

3. Linear and Nonlinear Transformations:

In Strategies B/C, the algorithm utilizes 4 S-boxes (Table A1, Table A2, Table A3 and Table A4) to achieve 8-bit nonlinear substitution. The S-box design employs a mapping mechanism to strengthen resistance against differential attacks. The linear transformation L implements negation solely via XOR operations, maximizing logical resource utilization.

To systematically describe the complete execution flow of the Adaptive Feedback Compensation Algorithm, Algorithm 1 outlines the iterative logic of dynamic parameter computation (*β* and *α*), strategy selection branches (Strategies A/B/C), and linear/nonlinear transformations. The workflow proceeds as follows:
**Algorithm 1:** Adaptive Feedback Compensation Algorithm (AFCA)
**Input:** 128-bit plaintext *X* = (*X*_0_, *X*_1_, …, *X*_7_), where *X*_i_ ∈ (*Z*_2_^16^)^8^**Output:** 128-bit ciphertext *Y* = (*Y*_0_, *Y*_1_, …, *Y*_7_)1. Initialize *β*_prev = 0, *α*_prev = 02. Split *X* into eight 16-bit words: [*X*_0_, *X*_1_, …, *X*_7_]3. **For** *i* = 0 to 7 **do**: a. Compute *β_i_*:  **if** *i* = 0: *β_i_* = 8 − bit_count (*x*_0_^(*i*)^) − bit_count (*x*_1_^(*i*)^)  **else:**    *β_i_* = 8 − bit_count (*x*_0_^(*i*)^) − bit_count (*x*_1_^(*i*)^) + *β*_prev − *α*_prev b. Compute *α_i_*:  *α_i_* is assigned a value based on which range *β_i_* falls into. c. Select encryption strategy:  **case** *α_i_*:   0 (Strategy A):     *Y_i_* = *X_i_*   [1, 4] (Strategy B):     *y*_0_ = *x*_0_(*^i^*)     *y*_1_ = *Sbox*[*α_i_*](*x*_1_^(*i*)^)     *Y*_i_ = (*y*_0_, *y*_1_)   [−4, −1] (Strategy C):     Flip the elements of *x*_0_^(*i*)^.     *y*_1_ = *Sbox*[|*α_i_*|](temp_x1)     *Y_i_* = −(temp_x0, *y*_1_) d. Update parameters: *β*_prev = *β_i_*, *α*_prev = *α_i_*4. **Return** *Y* = (*Y*_0_, *Y*_1_, …, *Y*_7_)

Appendix B validates the algorithm’s characteristics: it optimizes data balance and randomness across both simple and complex environments, preserves partial original data features, and demonstrates strong compatibility in joint encryption with other algorithms. The modular design of AFCA ensures compatibility with multi-stage cryptographic frameworks, which is essential for complex applications like multi-user MDI-QKD systems [17], where both randomness quality and processing flexibility are critical. However, in specific scenarios (e.g., specialized environments or decryption-oriented applications), the algorithm’s improvement effects may fall below expectations.

## 3. Analysis of Post-Processing Algorithms

To validate the optimization effect of the AFCA on quantum random number sequences, this section systematically compares original data from continuous/discrete-type QRNGs with data processed by the algorithm. Randomness tests were conducted in compliance with NIST SP 800-22 [18] and GM/T 0005 [19] (Chinese National Cryptographic Standards) to ensure comprehensive validation, with a focus on evaluating the algorithm’s performance in balance enhancement and feature preservation. Results demonstrate that the algorithm dynamically compensates frequency deviations without significantly altering the statistical properties of original sequences, while maintaining strong compatibility with existing post-processing methods such as the SM4. This provides critical technical support for designing multi-stage post-processing architecture.

### 3.1. Analysis of Continuous-Type QRNG Post-Processing Algorithm

**Figure 1:**
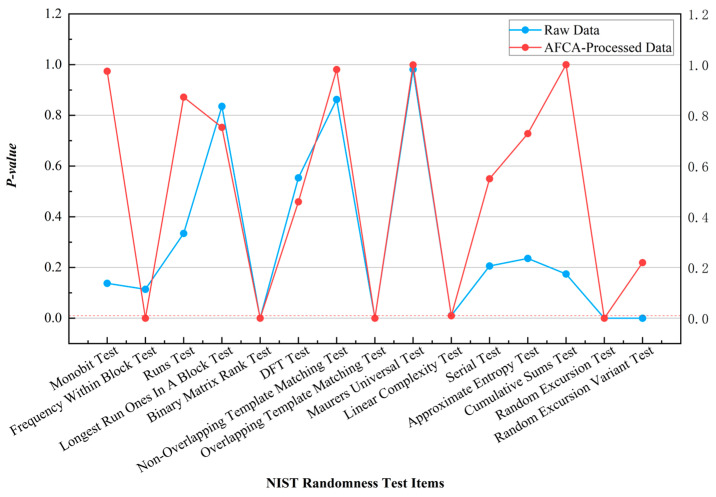
Comparison of NIST test results for continuous-type QRNG data processed by the AFCA.

Comparative results of NIST randomness tests for original continuous-type QRNG data and AFCA-processed sequences are shown in Figure 1. In the NIST suite, the original data achieved a 73.3 percent pass rate across 15 subtests. Notably, the Monobit Test *p*-value improved from 0.1376 to 0.9743, indicating that the algorithm effectively eliminated 0/1 bias by dynamically adjusting bit distribution weights. In the Frequency Within Block Test, the original *p*-value of 0.1140 decreased to 1.02 × 10^−5^ post-processing. While the *p*-value reduction is significant, its magnitude remains within statistically acceptable fluctuation ranges. This result highlights the algorithm’s capability to correct local frequency deviations without substantially perturbing the sequence’s global statistical properties, demonstrating its dual capacity for global balance optimization and original sequence characteristic preservation.

**Figure 2:**
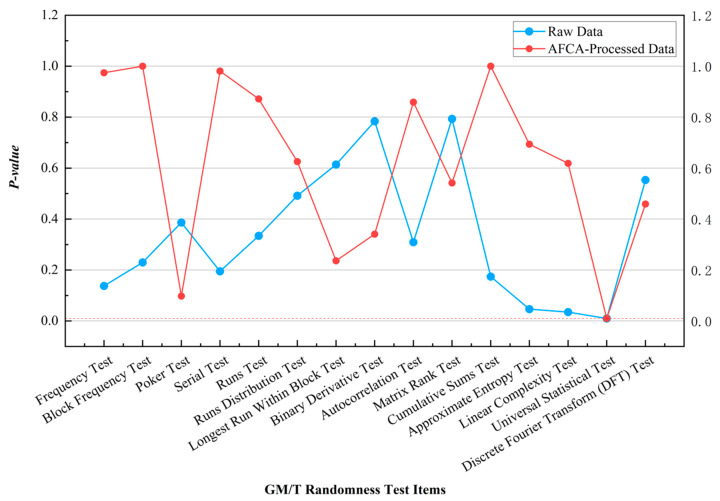
Comparison of GM/T standards testing results for continuous-type QRNG data processed by the AFCA.

In GM/T standards testing, the results are shown in Figure 2. The *p*-values of the Frequency Test and Runs Test improved from 0.1376 and 0.3343 to 0.9743 and 0.8718, respectively, validating the effectiveness of the feedback compensation mechanism in enhancing global balance and short-range independence. This improvement stems from the nonlinear confusion introduced by the S-box substitution in the algorithm for short-period patterns. Notably, the *p*-value of the Longest Run Within Block Test slightly decreased from 0.8355 to 0.7527, which may relate to the algorithm’s conservative processing strategy for long consecutive bit sequences, though the result remains within the acceptance threshold.

Additionally, the algorithm exhibited negligible impact on certain test items. For instance, the Universal Statistical Test failed for both raw and processed data due to insufficient data volume requirements for testing, rather than inherent flaws in the algorithm. Similarly, the *p*-value of the Discrete Fourier Transform (DFT) Test showed minor fluctuations from 0.5536 to 0.4590, further demonstrating that the algorithm avoids excessive perturbation of the original sequence’s characteristics. This selective optimization strategy not only rectifies critical balance deficiencies but also preserves the sequence features of the raw data, laying a foundation for the multi-layered application of encryption algorithms.

### 3.2. Analysis of Post-Processing Algorithm for Discrete-Type QRNG

**Figure 3:**
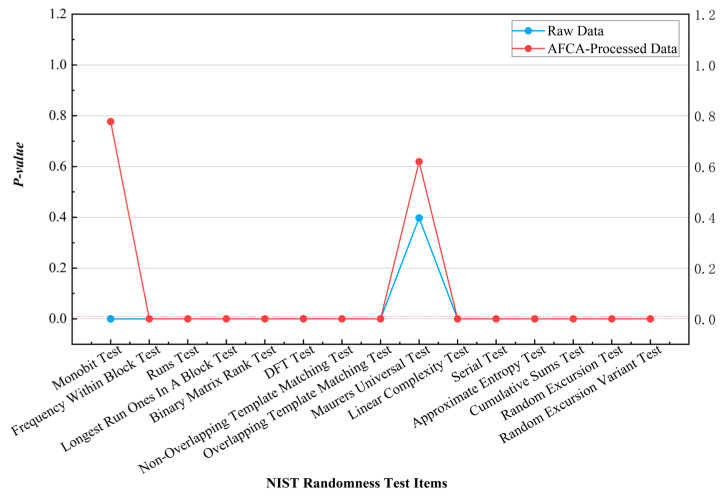
Comparison of NIST test results for discrete-type QRNG data processed by the AFCA.

**Figure 4:**
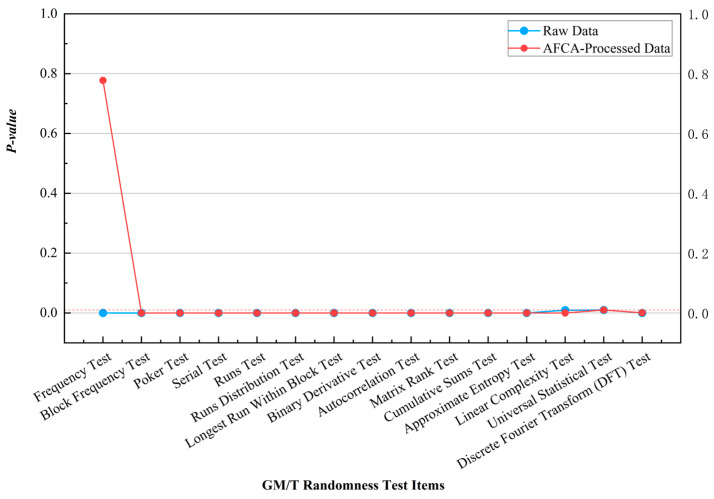
Comparison of GM/T standards testing results for discrete-type QRNG data processed by the AFCA.

The raw data of discrete-type QRNGs exhibit limited performance in randomness testing due to interference from single-photon detector dead-time effects and thermal drift, as illustrated in the comparative results shown in Figure 3 and Figure 4. Taking the NIST tests as an example, the severely imbalanced Monobit Test (*p* = 3.73 × 10^−5^) in the raw data improved to a *p*-value of 0.7773 after processing, indicating that the algorithm reduces the 0/1 distribution deviation from 7.9 percent to 0.5 percent through its parameter feedback mechanism. Similar results were observed in GM/T standards testing, where the algorithm enhanced sequence balance while retaining the limited performance of raw data in other test items.

In summary, the AFCA demonstrates advantages in “targeted correction” and “compatibility preservation” across different QRNG types. Its optimization of frequency deviations and short-range patterns exhibits universality, while its “inert processing” of other items (e.g., DFT tests) avoids entropy loss caused by excessive post-processing. These characteristics enable the algorithm to function independently as a lightweight post-processing module or collaborate with other post-processing algorithms (e.g., hash functions, block ciphers), providing a flexible technical pathway for quantum information productization across diverse scenarios.

While NIST SP 800-22 and GM/T 0005 provide standardized cryptographic evaluation, recent studies indicate that comprehensive randomness assessment should incorporate multi-dimensional validation [20]. Advanced test suites (e.g., TestU01, PractRand) offer enhanced sensitivity to long-range correlations and non-uniform patterns, particularly critical for entropy sources with subtle deviations. Future implementations may integrate such extended verification to further validate AFCA’s robustness.

## 4. Comparative Analysis of Post-Processing Algorithms

To comprehensively evaluate performance differences among post-processing algorithms, this study reproduced the hash-based SM3 and von Neumann Correction Method using Python 3.13, conducting comparative experiments on raw random sequences generated by continuous type QRNGs. Quantitative analysis of NIST SP 800-22 test results reveals distinct characteristics of different algorithms in randomness optimization.

In the engineering implementation of the SM3 algorithm, adaptive modifications were made to resolve its native block compatibility issues with arbitrary-length input data: input data is segmented into fixed 256-bit blocks, and a padding-truncation mechanism ensures output length consistency. The modified algorithm retains the hash function’s strong confusion capability while significantly improving processing efficiency, establishing a quantifiable benchmark for post-processing speed comparisons.

**Figure 5:**
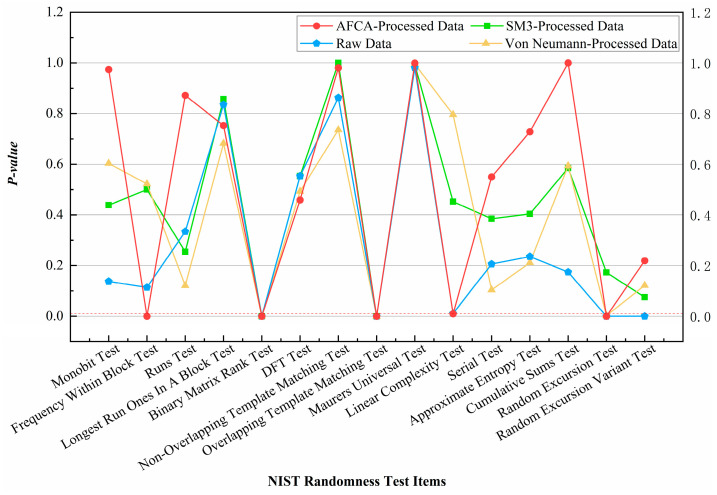
Comparison of NIST test results for continuous-type QRNG data optimized by post-processing algorithms.

Experimental data indicate that raw continuous-type QRNG sequences exhibit high randomness without post-processing, though the Binary Matrix Rank Test and Overlapping Template Matching Test were incomplete due to insufficient sample sizes (see Figure 5).

Comparison of NIST results in Figure 5 demonstrates that the AFCA excels for continuous-type QRNGs, improving the Monobit Test *p*-value from 0.138 to 0.974, with the differences between original and processed *p*-values for other test items constrained to ≤0.15 for reductions, while permitting unrestricted improvements when values increase. This confirms its ability to enhance balance while preserving original sequence features. Table 2 presents the key performance metrics of AFCA and competing algorithms in the tests. The reduced data discard rate of AFCA not only preserves entropy but also aligns with the cost-optimization requirements of large-scale QKD networks [21], where resource efficiency directly impacts operational feasibility.

The AFCA delivers optimal performance in high-entropy scenarios, with its dynamic feedback mechanism enabling cascaded application with the SM4 or other post-processing methods (Appendix B presents an example of its cascading use with SM4). The real-time statistical feedback mechanism in AFCA is particularly suited for dynamic environments, such as free-space QKD with modulating retroreflectors [22], where channel fluctuations demand rapid algorithmic responses. In contrast, SM3 and von Neumann methods, which entirely disrupt original sequence features, struggle to integrate into multi-stage processing architectures. Notably, the adaptive algorithm’s limitations in low-entropy source processing stem from its reliance on input sequence statistical features—when raw entropy source quality is insufficient, the adjustment range of parameters $\alpha_{i}$ fails to compensate for systemic deviations. Experimental results show that when processing the same amount of raw data, AFCA is 18% faster than SM3 and 71% faster than the von Neumann architecture.

To statistically validate AFCA’s core capability of selectively correcting 0/1 imbalance, we conducted focused Monte Carlo simulations using the Monobit Test. A 10,000-bit raw quantum sequence was processed through different algorithms and segmented into independent 100-bit subsequences for individual testing.

**Figure 6:**
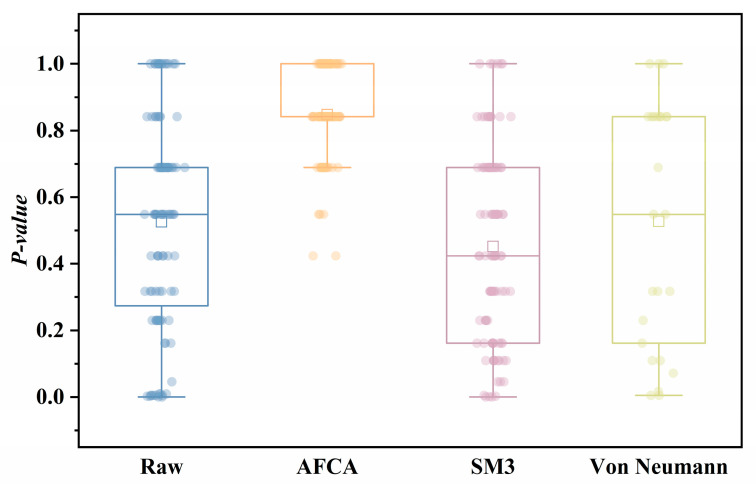
Statistical distribution of Monobit Test *p*-values: Comparative analysis of balance correction efficacy.

Figure 6 and Table 3 reveal AFCA achieves 100% pass rate, significantly outperforming raw data (88%), SM3 (94%), and von Neumann (92%) methods. Its median *p*-value of 0.85 demonstrates exceptionally reliable balance correction, while the minimal IQR span of 0.16 with 75% of data concentrated in the high-confidence interval (0.84–1.00) indicates outstanding stability. In contrast, SM3 shows unstable correction with the lowest median *p*-value (0.45) and largest IQR distribution (0.53), while von Neumann’s high data discard rate substantially reduces usable samples (only 25 segments), imposing impractical application costs.

## 5. Conclusions

We proposed the Adaptive Feedback Compensation Algorithm to enhance the post-processing of quantum random number generation. AFCA dynamically adjusted processing intensity based on real-time statistical feedback, enabling precise 0/1 balance correction (e.g., the Monobit Test *p*-value improved from 0.1376 to 0.9743 for continuous QRNGs). It employed a selective encryption strategy, retaining more than 50 percent of the original bits while applying nonlinear transformations exclusively to high-deviation regions, significantly reducing 0/1 distribution deviation (e.g., from 7.9 percent to 0.5 percent for discrete QRNGs). NIST results demonstrated that AFCA offered superior balance enhancement capabilities compared to traditional algorithms and effectively reduced the data discard rate. Furthermore, AFCA’s characteristic of retaining original data enabled cascade operations with other algorithms, providing notable compatibility.

The AFCA operates within inherent constraints of local post-processing: (1) Its effectiveness depends on the underlying entropy source quality, as severely biased inputs may exceed the compensation range of adaptive parameters. (2) Real-time computation of *β_i_*/*α_i_* and selective encryption requires moderate computational resources, making ultra-lightweight implementations an optimization target. (3) Like all post-processing, it cannot create entropy but redistributes existing entropy—physical-layer enhancements remain crucial for fundamental bias mitigation.

Future work will explore hybrid approaches combining entropy-source monitoring [23] with AFCA’s dynamic compensation, and evaluate performance under expanded test suites.

## Figures and Tables

**Table 1:** Notations and definitions.

Notations	Definitions
$Z_{2}^{m}$	Binary sequence with bit-length *m*
${\left(Z_{2}^{m}\right)}^{n}$	Set of *n* binary sequences each with bit-length *m*
word	16-bit word (block/string)
Substitution Box (*Sbox*)	Fixed input-output substitution, denoted as *Sbox*()
⊕	8-bit/16-bit XOR operation

**Table 2:** Performance comparison of post-processing algorithms.

Post-Processing Algorithm	Data Discard Rate	Monobit Test	Runs Test	Non-Overlapping Template Matching Test	Approximate Entropy Test
AFCA	0%	0.97	0.87	0.98	0.73
SM3 [11]	0%	0.44	0.25	0.99	0.40
von Neumann [7]	75%	0.60	0.12	0.74	0.21

**Table 3:** Quantitative Comparison of Balance Enhancement Metrics Across Processing Algorithms.

Algorithm	Samples	Pass Rate (%)	Median *p*-Value	IQR (25–75%)
Raw	100	88	0.53	0.42 (0.27–0.69)
AFCA	99	100	0.85	0.16 (0.84–1)
SM3	99	94	0.45	0.53 (0.16–0.69)
Von Neumann	25	92	0.53	0.68 (0.16–0.84)

**Figure A1:**
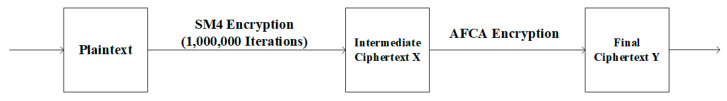
Joint algorithm operation flowchart.

## Data Availability

The original contributions presented in this study are included in the article. Further inquiries can be directed to the corresponding author.

## Appendix A. S-Box Configuration Data

This appendix provides the detailed S-box configuration data used in the nonlinear transformation τ of the Adaptive Feedback Compensation Algorithm. Table A1, Table A2, Table A3 and Table A4 correspond to different parameter values of $\alpha_{i}$ (±1 to ±4). Each table follows a 16 × 16 matrix format, where the row and column indices represent the high and low 4 bits of the input value, respectively. The cell entries denote 8-bit hexadecimal output values. Appendix A provides the S-box configuration data table used by the algorithm.

**Table A1:** S-box data $(\alpha_{i}\;=\;\pm 1)$.

	0	1	2	3	4	5	6	7	8	9	A	B	C	D	E	F
0	08	81	42	23	14	85	46	27	18	89	4A	2B	1C	8D	4E	2F
1	14	19	16	1B	15	1D	17	37	1A	39	9A	5B	3C	9D	5E	3F
2	22	29	26	33	2C	35	A6	67	2C	39	6A	6B	3C	AD	6E	3F
3	31	35	3A	3B	3C	3D	3E	B7	39	3D	3E	7B	BC	BD	7E	BF
4	48	43	46	C3	46	65	56	C7	4C	69	5A	CB	6C	5D	CE	CF
5	54	59	53	57	55	57	57	77	5A	5D	5B	DB	DC	7D	7E	7F
6	62	65	6A	6B	6C	6D	6E	E7	69	6B	6E	EB	7C	ED	EE	EF
7	71	73	76	77	76	77	77	F7	7C	7D	7B	7F	7D	7F	7F	FF
8	88	89	83	A3	85	A5	96	C7	8A	99	CA	CB	9C	AD	AE	AF
9	94	95	9A	9B	9C	97	9E	B7	99	9B	9E	DB	9E	BD	DE	BF
A	A2	A3	A6	A7	A6	AD	A7	E7	AC	AD	AB	EB	AD	BD	BE	EF
B	B1	B9	B3	BB	B5	B7	BE	BF	BC	BB	BE	BF	BE	BF	BF	FF
C	C8	C5	CA	C7	CC	CD	C7	D7	CA	CD	CB	EB	CD	ED	EE	DF
D	D4	D3	D6	DB	D6	D7	DE	DF	D9	DB	DE	DF	DE	DF	DF	FF
E	E2	E9	E3	E7	E5	ED	E7	EF	EA	ED	EB	EF	ED	EF	EF	FF
F	F1	F5	FA	FB	FC	F7	FE	FF	F9	FB	FE	FF	FE	FF	FF	FF

**Table A2:** S-box data $(\alpha_{i}\;=\;\pm 2)$.

	0	1	2	3	4	5	6	7	8	9	A	B	C	D	E	F
0	41	89	2A	17	85	35	66	F8	C8	99	AA	F4	5C	F2	F1	D7
1	D0	71	3A	EC	1E	EA	E9	3F	9C	E6	E5	DB	E3	7D	7E	DF
2	25	2D	2B	DC	2E	DA	D9	B7	2B	D6	D5	7B	D3	BD	7E	EF
3	36	CE	CD	3F	CB	BD	77	F7	C7	BD	BE	FB	7E	FD	FE	FF
4	4A	4B	4E	BC	47	BA	B9	E7	D8	B6	B5	EB	B3	7D	DE	7F
5	53	AE	AD	5F	AB	5F	5F	F7	A7	5F	5F	FB	5F	FD	FE	FF
6	6C	9E	9D	6F	9B	6F	6F	F7	97	6F	6F	FB	6F	FD	FE	FF
7	8F	7D	7E	7F	77	7F	7F	FF	7E	BF	7F	FF	7F	FF	FF	FF
8	85	8D	87	7C	87	7A	79	B7	8E	76	75	EB	73	DD	BE	EF
9	93	6E	6D	9F	6B	9F	9F	9F	67	9F	9F	FB	9F	FD	FE	FF
A	AC	5E	5D	AF	5B	AF	AF	F7	57	AF	AF	FB	AF	FD	FE	FF
B	4F	BD	B6	BF	BE	BF	BF	FF	BB	BD	BF	FF	BF	FF	FF	FF
C	C6	3E	3D	CF	3B	CF	CF	F7	37	CF	CF	FB	CF	FD	FE	FF
D	2F	D7	D7	DF	DE	DF	DF	FF	DB	DF	DF	FF	DF	FF	FF	FF
E	1F	E7	EB	EF	E7	EF	EF	FF	ED	EF	EF	FF	EF	FF	FF	FF
F	F5	FD	FE	FF	FD	FF	FF	FF	FE	FF	FF	FF	FF	FF	FF	FF

**Table A3:** S-box data $(\alpha_{i}\;=\;\pm 3)$.

	0	1	2	3	4	5	6	7	8	9	A	B	C	D	E	F
0	98	39	3A	B3	CC	E5	6F	E7	78	E9	AE	DB	CE	BD	7E	7F
1	53	B9	F2	F3	F4	F5	F6	F7	D9	F9	FA	FD	FC	FD	FE	FF
2	E1	6D	F2	F3	A7	F5	F6	F7	EC	F9	FA	FD	FC	FD	FE	FF
3	3B	3F	3F	BF	3F	FD	7F	FF	3F	FD	BF	FF	BF	FF	FF	FF
4	63	59	F2	F3	E5	F5	F6	F7	7C	F9	FA	FD	FC	FD	FE	FF
5	F1	5F	5F	FB	5F	7F	F7	FF	5F	7F	FB	FF	DF	FF	FF	FF
6	6E	6F	6F	FB	6F	7F	F7	FF	6F	EF	FE	FF	EF	FF	FF	FF
7	7E	7F	7F	FF	7F	FF	FF	FF	7F	FF	FF	FF	FF	FF	FF	FF
8	9C	8F	8F	F3	AE	F5	F6	F7	DA	F9	FA	FD	FC	FD	FE	FF
9	D3	9F	9F	DF	9F	F7	BF	FF	9F	FB	BF	FF	FD	FF	FF	FF
A	B5	AF	AF	BF	AF	FD	F7	FF	AF	EF	EF	FF	BF	FF	FF	FF
B	BD	BF	BF	FF	BF	FF	FF	FF	BF	FF	FF	FF	FF	FF	FF	FF
C	D5	CF	CF	DF	CF	EF	FE	FF	CF	FD	FB	FF	FE	FF	FF	FF
D	DB	DF	DF	FF	DF	FF	FF	FF	DF	FF	FF	FF	FF	FF	FF	FF
E	E7	EF	EF	FF	EF	FF	FF	FF	EF	FF	FF	FF	FF	FF	FF	FF
F	F7	FF	FF	FF	FF	FF	FF	FF	FF	FF	FF	FF	FF	FF	FF	FF

**Table A4:** S-box data $(\alpha_{i}\;=\;\pm 4)$.

	0	1	2	3	4	5	6	7	8	9	A	B	C	D	E	F
0	0F	F1	F2	F3	F4	F5	F6	F7	F8	F9	FA	FB	FC	FD	FE	FF
1	1F	EE	ED	FD	EB	F7	BF	FF	E7	DF	EF	FF	7F	FF	FF	FF
2	2F	DE	DD	FE	DB	FB	7F	FF	D7	BF	DF	FF	EF	FF	FF	FF
3	3F	DF	EF	FF	7F	FF	FF	FF	BF	FF	FF	FF	FF	FF	FF	FF
4	BF	BE	BD	F7	BB	FD	EF	FF	B7	7F	BF	FF	DF	FF	FF	FF
5	5F	7F	BF	FF	DF	FF	FF	FF	EF	FF	FF	FF	FF	FF	FF	FF
6	6F	FB	F7	FF	FE	FF	FF	FF	FD	FF	FF	FF	FF	FF	FF	FF
7	7F	FF	FF	FF	FF	FF	FF	FF	FF	FF	FF	FF	FF	FF	FF	FF
8	8F	7E	7D	FB	7B	FE	DF	FF	77	EF	7F	FF	BF	FF	FF	FF
9	9F	FD	FB	FF	F7	FF	FF	FF	FE	FF	FF	FF	FF	FF	FF	FF
A	AF	FE	FD	FF	FB	FF	FF	FF	F7	FF	FF	FF	FF	FF	FF	FF
B	BF	FF	FF	FF	FF	FF	FF	FF	FF	FF	FF	FF	FF	FF	FF	FF
C	CF	F7	FE	FF	FD	FF	FF	FF	FB	FF	FF	FF	FF	FF	FF	FF
D	DF	FF	FF	FF	FF	FF	FF	FF	FF	FF	FF	FF	FF	FF	FF	FF
E	EF	FF	FF	FF	FF	FF	FF	FF	FF	FF	FF	FF	FF	FF	FF	FF
F	FF	FF	FF	FF	FF	FF	FF	FF	FF	FF	FF	FF	FF	FF	FF	FF

## Appendix B. Operational Examples and Result Analysis

This section implements the AFCA proposed in the paper using Python code. The main workflow is as follows: First, the input 128-bit hexadecimal data is divided into eight 16-bit word groups. The *β* and α parameters are dynamically calculated by counting the number of bit-1 in each word group. Based on the sign and range of the α value, three encryption strategies are selected (direct output, S-box nonlinear transformation, negation, and S-box composite transformation). The S-box corresponds to four preset substitution tables according to the absolute value of α. Finally, the processed eight word groups are reassembled into the encrypted output. The code fully restores the core dynamic feedback compensation mechanism of the algorithm through parameter computation, strategy branching, and bitwise operations, while maintaining readability via modular structure. The example below directly verifies the algorithm’s effectiveness.

To validate the practical performance of the AFCA, this section demonstrates the encryption process and randomness enhancement through two typical examples. The examples cover both basic scenario verification (independent algorithm operation) and complex scenario verification (joint algorithm operations).

1. Independent Algorithm Operation Example

For this example, the plaintext input is 01 23 45 67 89 AB CD EF FE DC BA 98 76 54 32 10, which features a symmetric hexadecimal sequence (ascending followed by descending order) and strictly balanced single-bit frequency (50 percent 0/1 distribution). This input is ideal for verifying the algorithm’s ability to enhance randomness while preserving original characteristics. After processing by the algorithm, the output ciphertext is 01 FE 45 67 89 AB CD E0 FE 01 BA 98 76 54 32 1F, with the detailed encryption workflow shown in Table A5.

**Table A5:** Independent algorithm operation workflow.

Group Index	Input Plaintext (X)	$\alpha_{i}$	$\beta_{i}$	Output Ciphertext (Y)	Hexadecimal Mapping
0	0000 0001 0010 0011	4	4	0000 0001 1111 1110	01 FE
1	0100 0101 0110 0111	0	0	0100 0101 0110 0111	45 67
2	1000 1001 1010 1011	0	0	1000 1001 1010 1011	89 AB
3	1100 1101 1110 1111	−4	−4	1100 1101 1110 0000	CD E0
4	1111 1110 1101 1100	−4	−4	1111 1110 0000 0001	FE 01
5	1011 1010 1001 1000	0	0	1011 1010 1001 1000	BA 98
6	0111 0110 0101 0100	0	0	0111 0110 0101 0100	76 54
7	0011 0010 0001 0000	4	4	0011 0010 0001 1111	32 1F

Table A5 visually demonstrates the processing of the AFCA under symmetric input through the independent operation example. It shows that the algorithm enhances randomness while preserving data balance and retaining certain original features after computation.

2. Joint Algorithm Operation Example

When the input plaintext is 01 23 45 67 89 AB CD EF FE DC BA 98 76 54 32 10, the intermediate ciphertext after 1,000,000 SM4 encryptions becomes 59 52 98 C7 C6 FD 27 1F 04 02 F8 04 C3 3D 3F 66. Finally, after adaptive feedback compensation processing, the output ciphertext is 59 53 98 C7 C6 C5 27 1D 04 F2 F8 F4 C3 35 3F 60. The computational steps are detailed in Table A6.

**Table A6:** Joint algorithm operation workflow.

Group Index	SM4 Intermediate Ciphertext (X)	$\alpha_{i}$	$\beta_{i}$	Output Ciphertext (Y)	Hexadecimal Mapping
0	0101 1001 0101 0010	1	1	0101 1001 0101 0011	59 53
1	1001 1000 1100 0111	0	0	1001 1000 1100 0111	98 C7
2	1100 0110 1111 1101	−3	−3	1100 0110 1100 0101	C6 C5
3	0010 0111 0001 1111	−1	−1	0010 0111 0001 1101	27 1D
4	0000 0100 0000 0010	4	6	0000 0100 1111 0010	04 F2
5	1111 1000 0000 0100	4	4	1111 1000 1111 0100	F8 F4
6	1100 0011 0011 1101	−1	−1	1100 0011 0011 0101	C3 35
7	0011 1111 0110 0110	−2	−2	0011 1111 0110 0000	3F 60

It can be observed that the intermediate ciphertext after 1,000,000 iterations of SM4 encryption exhibits significantly greater disorder compared to the input plaintext. After processing by the AFCA, the output ciphertext improves balance (the ‘1’ proportion increased from 48 percent to 50 percent) while retaining certain features of the intermediate ciphertext.
